# Variation in Complete Blood Count Reports Across US Hospitals

**DOI:** 10.1001/jamanetworkopen.2025.14050

**Published:** 2025-06-05

**Authors:** Lewis T. Go, Lucas T. Go, Madugodaralalage D. S. K. Gunaratne, Jithma P. Abeykoon

**Affiliations:** 1 Onalaska High School, Onalaska, Wisconsin; 2 Carleton College, Northfield, Minnesota; 3 Division of General Internal Medicine, Mayo Clinic, Rochester, Minnesota; 4 Division of Hematology, Mayo Clinic, Rochester, Minnesota

## Abstract

This cross-sectional study of laboratory test records from US hospitals compares the total types of complete blood count results recorded in academic and community settings.

## Introduction

Complete blood count (CBC) is the second most common laboratory test performed in the US. In 2023, Medicare Part B alone spent nearly $300 million for 37 million CBC tests.^1^ It is estimated that about 500 million CBC tests are performed annually in the US.^2^ A typical CBC report contains almost 2 dozen different values. Currently, physicians are burdened by the substantial amount of time spent in reviewing the electronic medical record (EMR) during each patient encounter. About a third of this time is devoted to medical record review, including checking laboratory test results.^3^ The 21st Century Cures Act provides patients immediate access to their health information. However, this has created more anxieties and queries on the part of patients, which are reflected by increased patient portal messaging, sometimes even before their clinicians could review the test results.^4^ Particularly relevant, compared with other specialists, oncologists have a greater burden of messages received per day from EMR, with a third of the messages being related to test results.^5^

Little is known about the actual CBC values reported by various hospitals in the US both in the academic and community settings. We hypothesize that there is a wide variation in CBC reporting across hospitals.

## Methods

After approval from the Mayo Clinic institutional review board, we conducted this cross-sectional study in accordance with the Strengthening the Reporting of Observational Studies in Epidemiology (STROBE) reporting guideline. We obtained a list of CBC values reported by various hospitals in the US using records from the Epic EMR Care Everywhere health information exchange from 2020 to 2023. For each hospital, we determined the city and state where it was located. Data were compared between academic and community hospitals using descriptive statistics.

## Results

**Figure 1.**
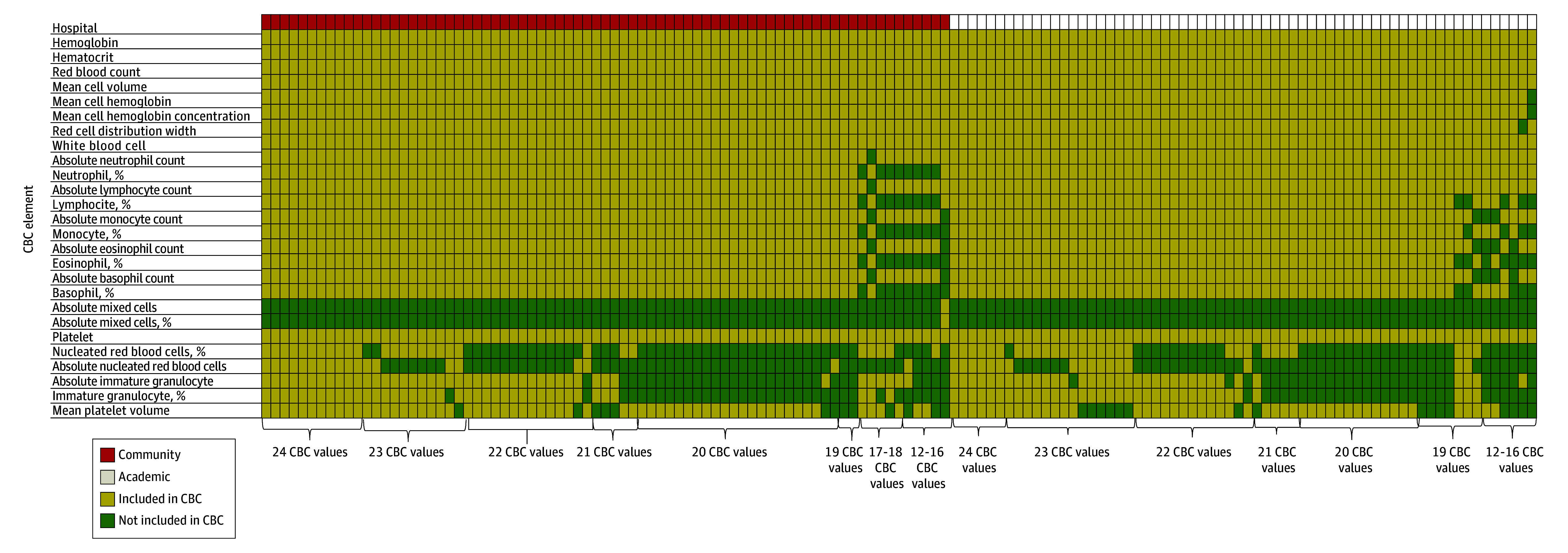
Type and Number of Complete Blood Count (CBC) Elements Reported Each column represents a single hospital and each row an element of CBC.

**Figure 2.**
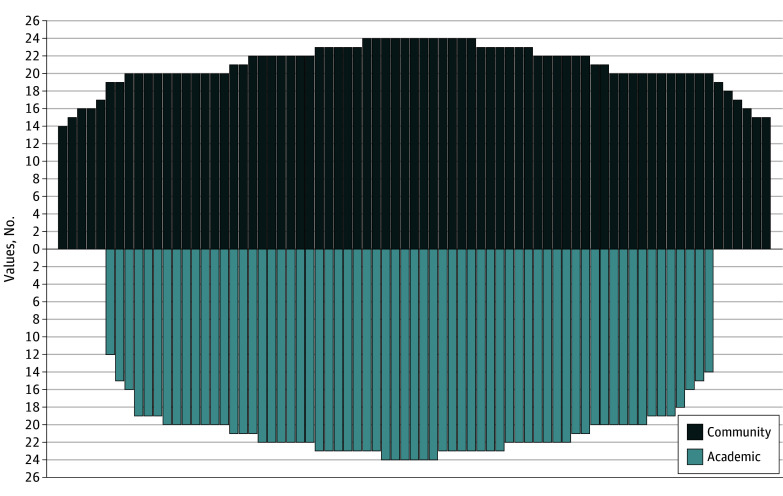
Comparison of the Number of Complete Blood Count Values by Hospital Type

We collected CBC values from 139 hospitals across 43 states and 102 cities (Figure 1). Fifty-four percent were community hospitals while 46% were academic hospitals. The median number of values reported by all hospitals was 21 (range, 12-24). This was not significantly different between academic (median 22 [range, 12-24]) and community hospitals (median 21 [range, 14-24]) (Figure 2). The distribution of hospitals according to the number of CBC values reported were: 12 to 16 (11 [8%]), 17 to 19 (14 [11%]), 20 (35 [25%]), 21 (10 [7%]), 22 (27 [19%]), 23 (25 [18%]), and 24 (17 [12%]). A substantial minority of the hospitals did not report the percentages of white blood cell differential count (13 [9%]) or mean platelet volume (29 [21%]). Absolute nucleated red blood cell and immature granulocyte counts were reported frequently, by 36 (26%) and 80 hospitals (58%), respectively. However, the frequency of reporting these 4 values were similar between academic and community hospitals.

## Discussion

We found substantial variations in CBC values reported across US hospitals. While most hospitals reported more than 20 values, twice as many values as the hospitals reporting the least number of values, 1 in 5 hospitals reported less than 20 values. This suggests that some of these values are either redundant or unnecessary, eg, the percentages of white blood cell differential count, mean platelet volume, nucleated red blood cell count, and immature granulocyte count. Our study is limited by the possibility that information available via EPIC Care Everywhere may differ from what is available to the local hospital. A 2023 report^6^ proposed 3 distinct abridged CBC reporting options, each for a specific purpose: CBC-WBC (5 values for periodic health examinations), CBC-Diff (9 values for acute illnesses), and CBC-Plus (10 values for hematology patients) as a potential solution. There is a huge opportunity to simplify and standardize CBC reporting. The foreseeable benefits include reducing EMR clutter and time spent in reviewing laboratory test results, minimize physician burnout and distraction, as well as decrease patient concerns and queries.

## Notes

Supplement.
Data Sharing Statement
