# Supplementary material for: Variation in Complete Blood Count Reports Across US Hospitals
Source: JAMA Netw Open. 2025 Jun 5;8(6):e2514050. doi: 10.1001/jamanetworkopen.2025.14050 (PMC12142446; doi:10.1001/jamanetworkopen.2025.14050)
Supplement: Supplement. — Data Sharing Statement [file jamanetwopen-e2514050-s001.pdf]

Data Sharing Statement

Go. Variation in Complete Blood Count Reports Across US Hospitals. *JAMA Netw Open*. Published June 05, 2025. doi:10.1001/jamanetworkopen.2025.14050

Data
Data available: Yes
Data types: Data (not involving human participants)
How to access data: By emailing Abeykoon.jithma@mayo.edu
When available: With publication

Supporting Documents
Document types: Statistical/analytic code
How to access documents: By emailing Abeykoon.jithma@mayo.edu
When available: With publication

Additional Information
Who can access the data: By emailing Abeykoon.jithma@mayo.edu
Types of analyses: or any purpose or for a specified purpose
Mechanisms of data availability: By emailing Abeykoon.jithma@mayo.edu
Any additional restrictions: None
